# A combination of inhibitors of glycolysis, glutaminolysis and *de novo* fatty acid synthesis decrease the expression of chemokines in human colon cancer cells

**DOI:** 10.3892/ol.2020.11303

**Published:** 2020-01-15

**Authors:** Alejandro Schcolnik-Cabrera, Guadalupe Dominguez-Gómez, Alma Chávez-Blanco, Marisol Ramírez-Yautentzi, Rocío Morales-Bárcenas, José Díaz-Chávez, Lucía Taja-Chayeb, Alfonso Dueñas-González

Oncol Lett 18: 6909-6916, 2019; DOI: 10.3892/ol.2019.11008

Subsequently to the publication of this paper, the authors have realized that the name of the sixth listed author, José Díaz-Chávez, was spelt incorrectly (it appeared as “José Chávez-Díaz” in print). The corrected author list, as it should have appeared in the paper, is shown above. The authors regret that the name of the sixth author on the paper was spelt incorrectly, and apologize to the readers for any inconvenience caused.

